# Exploring Spatiotemporal Accessibility of Urban Fire Services Using Real-Time Travel Time

**DOI:** 10.3390/ijerph18084200

**Published:** 2021-04-15

**Authors:** Yuehong Chen, Yuyu Li, Guohao Wu, Fengyan Zhang, Kaixin Zhu, Zelong Xia, Yu Chen

**Affiliations:** 1 College of Hydrology and Water Resources, Hohai University, Nanjing 210098, China; chenyh@lreis.ac.cn (Y.C.); liyuyu@hhu.edu.cn (Y.L.); wgh.hhu.edu.cn@hhu.edu.cn (G.W.); zhangfengyan@hhu.edu.cn (F.Z.); zhukaixin@hhu.edu.cn (K.Z.); 2 School of Urban Resource and Environment, Jiangsu Second Normal University, Nanjing 210013, China; xzl630415@163.com; 3 School of Geography, Nanjing Normal University, Nanjing 210023, China; 4 Jiangsu Center for Collaborative Innovation in Geographical Information Resource Development and Application, Nanjing 210023, China

**Keywords:** spatiotemporal accessibility, fire station, historical fire incidents, real-time travel time

## Abstract

The accessibility of urban fire services is a critical indicator in evaluating fire services and optimizing fire resource allocation. However, previous studies have mainly concentrated on measuring the spatial accessibility of fire services, and little, if any, consideration has been paid to exploring the spatiotemporal dynamics of the accessibility of urban fire services. Therefore, we used real-time travel time to extend an existing spatial accessibility method to measure the spatiotemporal accessibility of fire services in a case study of Nanjing, China. The results show that (1) the overall accessibility of fire incidents and fire stations in Nanjing, China, is uneven, with relatively high accessibility in the southwest and northeast of the city center; (2) the number of fire incidents with low-level accessibility apparently increases in rush hours (i.e., 07:00–09:00 and 17:00–19:00 h) in the southeast and north of the city center, and the fire incidents with medium-level and high-level accessibility easily change to lower levels under the influence of traffic congestion, with fire incidents with medium-level accessibility being affected the most; (3) the accessibility of fire stations changes over time with an obvious W pattern, with lower accessibility during rush hours than at other times, and several fire stations in the city center present an asymmetric W pattern; (4) the accessibility decline ratio for fire stations in rush hours is greater in the city center than in urban suburbs, and the decline ratios are strongly related to the travel time increase and the percent increase in uncovered fire incidents during rush hours. The results and findings demonstrate that our method can be used to explore the spatiotemporal dynamics of the accessibility of fire services, and so can guide policymakers in improving fire services.

## 1. Introduction

Fire is one type of disaster that produces serious harm and frequently occurs in modern society, which can cause physical damage and injures, economic losses, and environmental problems [1,2,3]. In 2019, a total of 233,000 fires were reported in China, with 1335 deaths and 837 injured [4]. As one of the important public services provided by governments, fire services (i.e., fire stations) can protect people, property, and the environment from fires and other emergencies [5]. The rational spatial deployment of fire services is essential for enhancing the accessibility to fire incidents to fire services, which can reduce the damage caused by fire incidents by shortening emergency response times [6,7,8]. Measuring the accessibility to fire services is therefore critical for governments to allocate fire rescue resources to ensure the adequate accessibility of fire services [6,9].

Accessibility can be viewed as the convenience of spatial elements in reaching the destination (e.g., public services or human activities) [10,11], and it includes spatial and nonspatial accessibility [12,13]. Spatial accessibility has been widely used in a variety of public services, and the most frequently used model is the two-step floating catchment area (2SFCA) model [14]. The 2SFCA method, introduced by Radke and Mu [14], comprehensively considers the potential demand, supply capacity, and geographical impedance between demand and supply. The 2SFCA method and its extensions have been adopted to measuring the spatial accessibility of healthcare services [15,16,17], park green space [18,19], job opportunities [20,21], transportation [22,23], and fire services [6,7,9,24]. Recently, real-time travel time has been used to examine the spatial accessibility of public services over time [17,19,21,23]. However, little, if any, consideration has been given to exploring the spatiotemporal dynamics of the accessibility of urban fire services.

To this end, in this study, we extended our previous method (fixed-coverage-based 2SFCA (FC2SFCA) [6]) to measure the spatiotemporal accessibility of fire services in a case study area in Nanjing, China, and the accessibility difference was explored both in space and time. The impact of traffic congestion on the accessibility of fire services was analyzed. In addition, suggestions are provided for policymakers to improve the fire services in the case study area. The contributions of this study include: (1) real-time travel time is introduced to investigate the spatiotemporal dynamics of the accessibility of fire services, (2) the novel spatiotemporal FC2SFCA method is developed to measure the spatiotemporal accessibility of fire services, and (3) the proposed method is implemented in a case study to explore the spatiotemporal pattern of the accessibility of fire services.

## 2. Literature Review

The 2SFCA method has been extended in four aspects [25,26]: (1) adding spatial decay weights [27,28,29]; (2) adjusting the size of catchment sizes [30,31]; (3) using the competition of supply and demand [32]; and (4) considering multiple travel behaviors from supply to demand [33,34]. These extensions have been applied in various public services [25,26,35,36], including fire services [6,7,9,24]. Min, Kim and Lee [24] employed 2SFCA to measure the spatial accessibility of fire services in Dallas, Texas, USA, and found it was significantly associated with unintentional residential fire-related injuries. Kiran, Corcoran and Chhetri [7] drew on small-area population forecasts and applied the enhanced 2SFCA method to calculate the fire service accessibility in Brisbane, Australia. Xia, Li, Chen and Yu [9] proposed an optimized 2SFCA method to measure the accessibility of urban fire services that incorporates both spatial and nonspatial factors. Mao, Chen, Wu, Huang, Yang and Xia [6] proposed a fixed-coverage-based 2SFCA (FC2SFCA) method to measure the accessibility of urban fire services under the spatial constraint of fire service coverage. However, most of these studies were limited to only mapping the spatial pattern of fire services accessibility and little effort has been devoted to measuring the accessibility of urban fire services over temporal changes. Urban traffic conditions temporally fluctuate, which can lead to the dynamic spatial impedance between fire stations and fire incidents in assessing the accessibility of fire services. Although fire engines are free from traffic light waiting time, nowadays, urban traffic congestion is increasingly worsening with the acceleration of urbanization [37,38], which has significantly prolonged the response time of fire services, especially during rush hour [39,40], further resulting in severe temporal fluctuation in accessibility [41]. Therefore, the spatiotemporal measurement of the accessibility of fire services is necessary for fire service agencies to better allocate fire resources and improve fire service accessibility.

In recent years, a variety of studies have adopted 2SFCA and its extensions to focus on the temporal change in the spatial accessibility of public services using real-time travel time, such as park green spaces [18,19], job opportunities [20,21], transportation [22,23], and especially healthcare services [15,16,17]. For instance, Jamtsho, Corner and Dewan [15] proposed the nearest-neighbor modified 2SFCA to analyze the accessibility of primary healthcare services from 2010–2013 in Bhutan both spatially and temporally. Chen, Wang, Jin, Xia, Xiao, Chen and Luo [16] employed the Gaussian 2SFCA method to calculated the elderly’s medical services accessibility in inner-city Wuhan, China, within a month, and presented a sensitivity analysis of the accessibility scores for several thresholds of time and different time intervals in one day. Xia, Song, Zhang, Song, Kanasugi and Shibasaki [17] presented a spatiotemporal accessibility model based on the enhanced 2SFCA method, which used a large-volume global positioning system (GPS) dataset to measure the spatiotemporal accessibility of emergency medical services in Tokyo, Japan. However, few studies investigated the spatiotemporal dynamics of the accessibility of fire services.

## 3. Materials and Methodology

### 3.1. Materials

**Figure 1:**
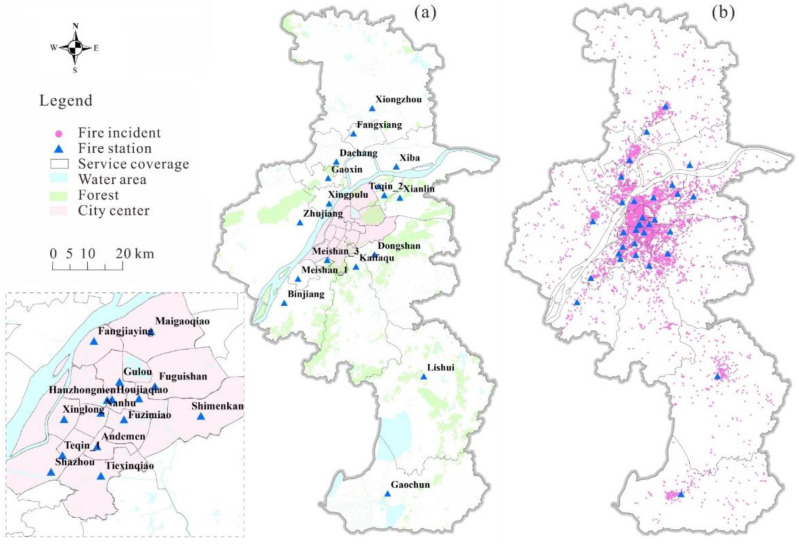
Study area and data in Nanjing, China: (**a**) the service coverage of fire stations; (**b**) historical fire incidents in 2015.

Nanjing is the capital of Jiangsu province and is located in the Yangtze River Delta, which is one of the most economically developed regions in China. In past years, Nanjing has experienced rapid urban expansion, with the rate of urbanization reaching 83.2% in 2019. At the same time, the number of urban fires increased from 2965 in 2012 to 8481 in 2015 [6]. Thus, Nanjing is a typical city with both rapid urbanization and an increase in urban fires in China, so it was selected as the case study area. Nanjing has 32 fire stations and their locations and service coverages are shown in Figure 1a. The number of firefighters and fire engines at each fire station and the 8481 historical fire incidents (Figure 1b) in 2015 were collected from the Nanjing Fire Department [42]. The firefighters and fire engines were used to calculate the supply capacity at each fire station [6,9]. For each fire incident, we recorded its location, the fire station involved in the rescue, the number of fire engines used in the rescue, etc. The number of fire engines used in the fire incident was used as the demand size [6]. For measuring the spatiotemporal accessibility of fire services, the fire incidents were employed as the demand nodes, whereas fire stations were used as the supply nodes.

### 3.2. Background of 2SFCA and FC2SFCA

The supply capacity, demand size, and the impedance between the supply facilities and demand nodes are three key factors in 2SFCA and its extensions in the measurement of accessibility. The supply capacity often represents the size of resources held by the supply facility for providing services. For instance, the number of firefighters and fire engines at a fire station can be used as the supply capacity of the fire station, as a fire station with larger capacity can provide sufficient firefighters and fire engines for handling larger fire incidents [6,9]. The demand size means the scale of service resources required by a demand node. For example, fire incidents with different sizes require different numbers of firefighters and fire engines. The impedance accounts for the geographical proximity between demand nodes and supply facilities. The traditional 2SFCA method needs two steps for measuring the spatial accessibility [14]. In the first step, all demand nodes are first searched within a given catchment area (e.g., a circle centered at a supply facility) of the supply facility and then the supply-to-demand ratio is computed for the supply facility using the supply capacity divided by the total demand nodes. The supply-to-demand ratio is usually considered as the service availability of the demand nodes. In the second step, the supply-to-demand ratios within a given catchment area of a demand node are summed up and regarded as the spatial accessibility of the demand node. For the supply-to-demand ratio calculation of fire services, however, the catchment area of a fire facility (i.e., fire station) cannot be directly used to search for the demand nodes because each fire station is usually responsible for fire incidents within its coverage area [6]. The FC2SFCA method therefore proposed using the coverage area of fire stations as the catchment area for measuring the accessibility of urban fire services [6]. Although FC2SFCA can effectively measure the spatial accessibility of fire services, it cannot present the accessibility of fire services over time.

### 3.3. Spatiotemporal (ST)-FC2SFCA

**Figure 2:**
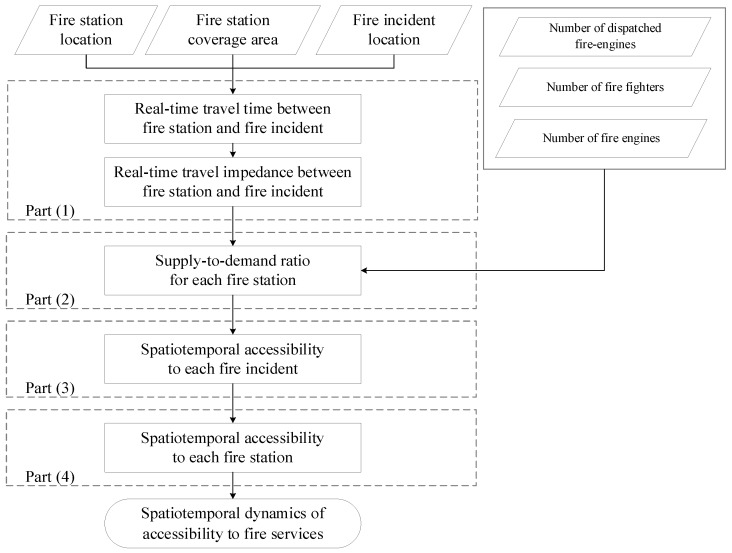
Flowchart of the spatiotemporal pattern of accessibility of fire services in Nanjing, China.

Recently, various works have adopted online map APIs to acquire real-time travel time to measure the spatiotemporal accessibility of public services, obtaining satisfactory performance [16,19,26]. Therefore, we used real-time travel time to extend the previous FC2SFCA in both the spatial and temporal dimensions as the spatiotemporal FC2SFCA (ST-FC2SFCA) for measuring the spatiotemporal accessibility of fire services. ST-FC2SFCA includes four main parts, as displayed in Figure 2:

(1) Calculate real-time travel impedance between a fire incident and its involved fire station. The real-time travel times between a fire incident as the origin and its involved fire station as the destination should be acquired from an online map API first. Then, the real-time impedance for each time interval is computed using a travel-time decay function.
(2) Compute the supply-to-demand ratio for each fire station. The numbers of fire fighters and fire engines at each fire station are combined to represent the supply capacity of the fire station. The covered fire incidents of a fire station and the dispatched fire engines in each fire incident are used to estimate the demand size of a fire station. With the supply capacity and demand size of a fire station as inputs, the spatiotemporal ratio of the fire station can be computed.
(3) Measure the spatiotemporal accessibility of fire incidents. The spatiotemporal accessibility of each fire incident is measured by the supply-to-demand ratio of its involved fire station and its real-time travel impedance.
(4) Measure the spatiotemporal accessibility of fire stations. The spatiotemporal accessibility of fire incidents covered by each fire station is used to measure the spatiotemporal accessibility of each fire station.

#### 3.3.1. Estimation of Real-Time Travel Impedance

Although fire engines do not need to wait for traffic lights, the increasingly worse urban traffic congestion, especially in daytime, severely prolongs the response times of fire engines [37,38]. The fluctuating traffic condition results in changes in the travel impedance between a fire station and a fire incident. The online map API can capture the real-time travel time between an origin and a destination at a time stamp. The AutoNavi Maps API [43], one of the biggest online maps in China, has been broadly adopted in measuring the accessibility of public services [6,16,26]. The effectiveness of online map API has been demonstrated for estimating accurate travel time of fire engines in previous fire studies [6,44]. Thus, the private car mode of the AutoNavi Maps API was employed to acquire the real-time travel time between a fire station to a fire incident. The impedance between a fire station and a fire incident can be measured by either travel time or travel distance using decay functions, such as Gaussian function, kernel density function, etc. [6,25]. Decay functions generate the gradually decreasing impedance weights with the increase in travel time or travel distance. These decay functions are more accurate than using original travel time and travel distance to capture the change in geographical impedance; they are widely used in various accessibility measurements [25]. Fire services are different from other public services (e.g., healthcare services) and a quick response time is necessary to handle fire incidents. Therefore, the travel time between a fire station and a fire incident was employed as the input of the decay function to measure the impedance in measuring the accessibility of fire services. With the real-time travel time as the input, the hybrid travel-time decay function developed in [6] was extended to measure the real-time travel impedance between a fire station and a fire incident for the subsequent measurement of spatiotemporal accessibility.

According to experience with traffic dynamics, the traffic condition in Nanjing, China, mainly fluctuates from 05:00–23:00 h, and travel is unimpeded at other times of day. Hence, we acquired the real-time travel time between a fire station and a fire incident every 10 min from 05:00–23:00 h of each day during 3 weeks (7–27 September 2020). That is, the real-time travel time for each fire incident was acquired once during each time interval of 10 min from the AutoNavi Maps API and each fire incident received 2268 real-time travel time values during the 3 weeks. We used 3 weeks to avoid the randomness of real-time travel time on 1 day. Generally, the time between 07:00–09:00 is the morning rush hour and the time from 17:00–19:00 h is the evening rush hour in Nanjing, China. For this, the real-time travel time during the 3 weeks of each fire incident was aggregated to the average real-time travel time every 2 h of one day, yielding nine averaged real-time travel time values for each fire incident. The nine averaged real-time travel time values were viewed as the final real-time travel time in 1 day for calculating real-time travel impedance. The real-time travel time $RT_{ij}\left(k\right)$ at time interval k from fire station i to fire incident j can be computed as

(1) $$RT_{ij}\left(k\right)=\frac{1}{L}\sum_{l=1}^{L}rt_{ij}\left(l\right)$$

where $rt_{ij}\left(l\right)$ is the real-time travel time at time stamp l during the three weeks, L=2268 is the total number of time stamps, and $k\in \left\{1,2,\ldots ,9\right\}$ is the number of time intervals of one day.

The hybrid travel-time decay function is extended in the temporal dimension to calculate the real-time travel impedance between a fire station i and a fire incident j by

(2) $$\omega_{ij}\left(k\right)=\left\{\begin{array}{cc} 1 & RT_{ij}\left(k\right)\leq 300s\\ (e^{-\frac{1}{2}{\left(\frac{RT_{ij}\left(k\right)-300}{900}\right)}^{2}}-e^{-\frac{1}{2}})/\left(1-e^{-\frac{1}{2}}\right) & 300s<RT_{ij}\left(k\right)\leq 1200s\\ 0 & RT_{ij}\left(k\right)>1200s \end{array}\right.$$

where $\omega_{ij}\left(k\right)$ is the real-time travel time at time interval k.

The hybrid travel-time decay function is a piecewise function that ensures shorter travel time generates a greater weight for the geographical impedance. For real-time travel time $RT_{ij}\left(k\right)\leq 300\;s$, the weight is set to 1 because Chinese national regulations stipulate firefighters should arrive at the fire incident place within 5 min after they receive the fire alarm. For real-time travel time $300s<RT_{ij}\left(k\right)\leq 1200\;s$, the weight decreases from 1 to 0 with the increase in travel time. For real-time travel time $RT_{ij}\left(k\right)>1200\;s$, the weight is set to 0, which means 20 min is regarded as the maximum catchment radius used in the case study area [6].

#### 3.3.2. Calculation of Supply-to-Demand Ratio of Fire Stations

The supply facility, the fire station, is different from other public facilities (e.g., hospitals, parks, transportation, etc.), which may be chosen freely when obtaining services. Instead, each fire station has a service coverage area, where only one fire station is responsible for the covered fire incidents except a very few mass fires. Therefore, we replaced the traditional circle catchment area with the coverage area (Figure 1a) as the catchment area of a fire station, and the covered fire incidents of each fire station were used to calculate the demand nodes for a fire station. Usually, larger urban fires need more fire engines and firefighters, which means the fire incident demands more fire services. The dispatched fire engines to a fire incident can therefore be considered as the demand size of the fire incident. Firefighters and fire engines are the crucial resources of fire stations, as firefighters must drive fire engines to the fire incident scene to extinguish the fire. The supply capacity of fire stations can be described by combining the two factors. Thus, we also employed the firefighters and equipped fire engines of a fire station to measure the supply capacity of the fire station, as used in previous study [6]. The supply-to-demand ratio $R_{i}$ of fire station i is calculated by

(3) $$R_{i}=\frac{S_{i}}{\sum_{j\epsilon i}D_{j}}$$

where $S_{i}$ is the supply capacity measured by the sum of firefighters and fire engines at fire station i, and $D_{j}$ is the number of dispatched fire engines for fire incident j within fire station i.

#### 3.3.3. Spatiotemporal Accessibility Measurement of Fire Incidents

The supply capacity of a fire station does not temporally change in 1 day during a short period and we assumed that the supply capacity of fire stations stays the same in one day. As historical fire incidents did not record the time of day of the occurrence, we assumed that all fire incidents were distributed uniformly across time in a day. The accessibility to all fire incidents was measured over time. However, the travel impedance between a fire station as the supply node and a fire incident as the demand node severely fluctuates along the time of day. Therefore, the spatiotemporal accessibility of fire incidents as the real demand was mainly measured by combining the unchanged supply-to-demand ratio in Equation (3) and the real-time travel impedance in Equation (2). The spatial accessibility to fire incidents at each time interval was first calculated and then the nine accessibilities at different time intervals of 1 day were used to represent the spatiotemporal accessibility of fire incidents. As aforementioned, a fire station has a coverage area and a fire incident usually can obtain fire services from its covered fire station. Hence, the coverage area of a fire station was adopted to replace with the catchment area of a fire incident, and the spatiotemporal accessibility of a fire incident is computed by

(4) $$A_{j}\left(k\right)=\sum_{j\epsilon i}R_{i}\omega_{ij}\left(k\right)$$

where $A_{j}\left(k\right)$ is the spatiotemporal accessibility of fire incident j at time interval k in 1 day.

#### 3.3.4. Spatiotemporal Accessibility Measurement of Fire Stations

To investigate the accessibility fluctuation of fire stations over both space and time, we used the average spatiotemporal accessibility of fire incidents within a fire station to represent its spatiotemporal accessibility, which is calculated by

(5) $$F_{i}\left(k\right)=\frac{1}{N}\sum_{j=1|j\epsilon i}^{N}A_{j}\left(k\right)$$

where $F_{i}\left(k\right)$ is the spatiotemporal accessibility of fire station i at time interval k in 1 day.

## 4. Results

### 4.1. Spatiotemporal Accessibility of Fire Incidents

**Figure 3:**
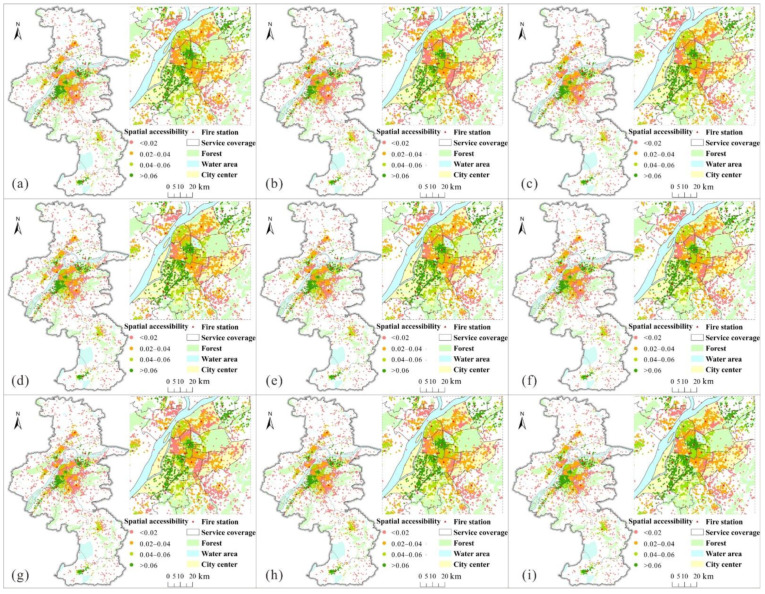
Spatiotemporal accessibility of fire incidents in Nanjing, China: (**a**) 05:00–07:00, (**b**) 07:00–09:00, (**c**) 09:00–11:00, (**d**) 11:00–13:00, (**e**) 13:00–15:00, (**f**) 15:00–17:00, (**g**) 17:00–19:00, (**h**) 19:00–21:00, and (**i**) 21:00–23:00.

Figure 3 shows the accessibility of fire incidents at different times of day. The accessibility scores are divided into four levels: low (<0.02), medium (0.02–0.04), high (0.04–0.06), and very high (>0.06), as shown in Figure 3. Figure 3 shows that fire incidents in the areas around fire stations and city center had relatively higher accessibility, especially the southwest and northeast of the city center, where many fire incidents with high- and very-high-level accessibility were distributed. In contrast, fire incidents with relatively lower accessibility were mainly distributed in the suburbs and the north and southeast of the city center. The main reason for fire incidents with higher accessibility is that these areas have relatively stronger firefighting supply capacity (e.g., Shazhou and Teqin_1), shorter travel distances in a relatively small coverage area, and better traffic conditions than other areas with lower accessibility. Figure 3b,g presents the accessibility of fire incidents in the morning and evening rush hours, respectively, indicating that the number of fire incidents with low-level accessibility significantly increased in rush hours compared with other times in the southeast and north of the city center, while the number of fire incidents with high- and very-high-level accessibility changed slightly in the city center over time. Specifically, the accessibility of many fire incidents in the Hanzhongmen, Fuzimiao, Maigaoqiao, Gulou, Shimenkan, and Dongshan fire stations changed from medium-level in non-rush hour to low-level in rush hour, suggesting these areas were heavily influenced by traffic congestion during rush hour. According to the Nanjing travel report in 2019 released by Nanjing Traffic Management Bureau, the most congested road, Yingtian Street Elevated, which crosses the coverage area of Fuzimiao fire station and the area of Hanzhongmen fire station, is located in the second-most-congested district in Nanjing. In addition, the number of fire incidents with different accessibility levels changed slightly in urban suburbs over time.

**Figure 4:**
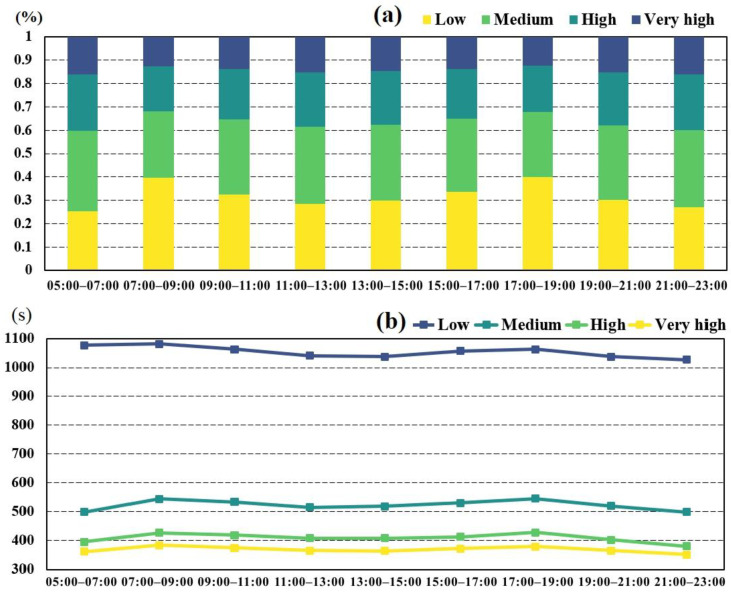
Change characteristics of fire incidents in 1 day. (**a**) Proportion of fire incidents and (**b**) average travel time of fire incidents at different accessibility levels.

Figure 4a,b displays the proportion and average travel time change of fire incidents at different accessibility levels in 1 day, respectively. Figure 4a shows that in morning and evening rush hours, the proportion of fire incidents with low-level accessibility notably increased, the proportion of medium- and high-level fire incidents decreased obviously, and the proportion of very-high-level fire incidents changed slightly. This suggests that fire incidents with medium- and high-level accessibility are more affected by traffic fluctuation than those with very-high-level accessibility. The proportion of fire incidents with low- and medium-level accessibility was over 60% for each time interval in 1 day. The proportion of fire incidents with low-level accessibility in rush hours increased by 10% to 40%, while the proportions of fire incidents with medium- and high-level accessibility both decreased more than 5% in morning rush hours. This means fire incidents with medium- and high-level accessibility easily change to lower accessibility levels induced by traffic congestion during rush hours. As for fire incidents with very-high-level accessibility, their proportion slightly fluctuated between 12.3–16.1% in 1 day. Figure 4b shows that fire incidents with lower accessibility required significantly more travel time and the travel time of fire incidents at the four levels increased in both morning and evening rush hours. Specifically, the travel time of fire incidents with low-level accessibility was over 1000 s in all intervals in 1 day, whereas the travel time of fire incidents with other accessibility levels was lower than 550 s. Compared with fire incidents with low-level accessibility, the travel time of fire incidents at other levels increased apparently during rush hours. The average travel time of fire incidents with medium-level accessibility increased the most during rush hours, which indicated that fire incidents with medium-level accessibility were the most affected by traffic congestion (see the clear peaks in Figure 4b).

### 4.2. Spatiotemporal Accessibility of Fire Stations

**Figure 5:**
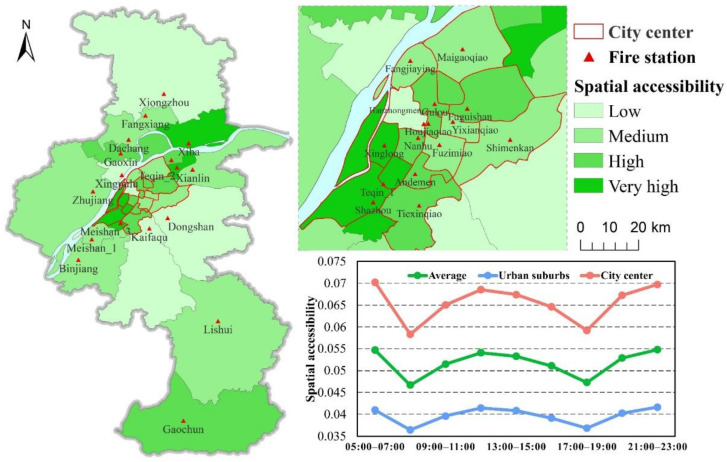
Spatiotemporal pattern of accessibility of fire stations in Nanjing, China.

In order to analyze the spatiotemporal accessibility of fire stations, the average accessibility to fire incidents within a fire station coverage area was calculated as the accessibility of the fire station, as displayed in Figure 5. The average accessibilities of fire stations in the whole Nanjing, city center, and suburbs were separately computed for different times of 1 day (Figure 5). As shown in Figure 5, the accessibility of fire stations in the city center generally was higher than those in suburbs. Among them, the accessibility of fire stations in the southwest and northeast of city center was relatively high. Even so, the overall accessibility of fire stations was low. The fire stations with low- and medium-level accessibility accounted for 15.6% and 37.5%, respectively, exceeding 50% of the total. As can be seen from the three lines of accessibility in Figure 5, the accessibility to fire stations changed over time in 1 day, showing an obvious W pattern in which the accessibility in morning and evening rush hours are lower than at other times. The accessibilities of fire stations in the whole of Nanjing, the city center, and suburbs were 13.41%, 15.97%, and 11.15% lower, respectively, than at other times, further indicating the accessibility of fire stations in the city center being more affected by traffic congestion than in suburbs.

**Figure 6:**
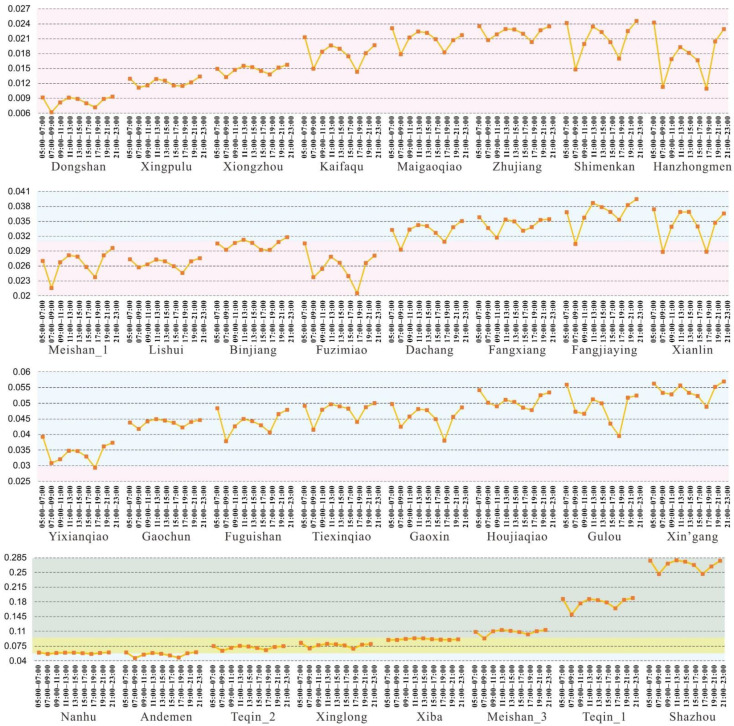
Accessibility of fire stations at different times of the day.

Figure 6 presents the spatiotemporal dynamics of the accessibility of each fire station in 1 day. Although the accessibility of each fire station showed a W pattern over time in 1 day, the accessibility decline of fire stations in rush hours was obviously different and several fire stations presented different declines during morning and evening rush hours. For example, Hanzhongmen, Fuzimiao, Shimenkan, Kaifaqu, and Dongshan fire stations showed the largest accessibility decrease in the morning and evening rush hours and their accessibility change in a W pattern over time was particularly obvious, while the accessibility of Nanhu and Xiba fire stations declined slightly in rush hours. Hanzhongmen, Fuzimiao, and Shimenkan fire stations are located in the city center and their accessibility was severely affected by the morning and evening commutes. As for Kaifaqu and Dongshan fire stations, the high number of residential buildings in the southeast periphery of the city center led to a large number of commuters, which resulted in an obvious accessibility decline during rush hours. Xiba fire station had a few fire incidents, causing a slight accessibility difference between rush hours and other times. Although Nanhu fire station is located in the city center, its coverage area is very small, so the accessibility declined only slightly during rush hours. Furthermore, several fire stations presented an asymmetric W pattern of accessibility over time. For instance, Fuzimiao and Gulou fire stations experienced a higher decline in evening rush hours than in morning rush hours. Fangjiaying fire station showed the opposite. Fuzimiao and Gulou fire stations are located in the most central areas of Nanjing in which office buildings and other work places are concentrated. Thus, road congestion in evening rush hours is heavier than that in morning rush hours, which in turn causes a greater accessibility decline for the two fire stations in evening compared to in morning rush hours. There are many residential buildings near Fangjiaying fire station, and a large commute demand occurs during morning rush hours, creating its apparent accessibility decline in morning rush hours.

### 4.3. Accessibility Decline Ratio of Fire Stations during Rush Hours

**Figure 7:**
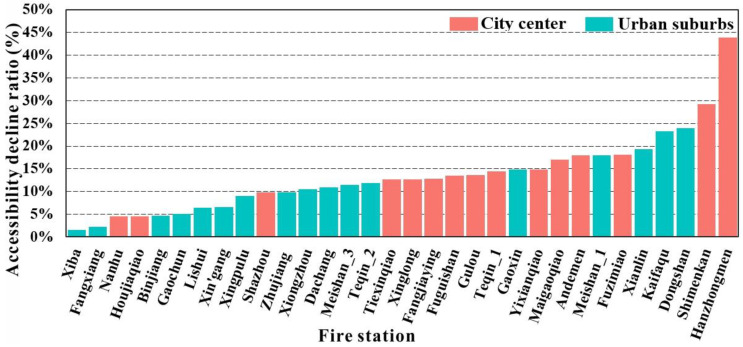
Accessibility decline ratio of fire stations during rush hours.

Figure 7 shows the accessibility decline ratio of fire stations during rush hours in comparison with the accessibility during non-rush hours in 1 day. We found that the accessibility decline ratio of fire stations in the city center was greater than that in the urban suburbs; namely, the decline ratio in the city center was 15.97% while being 11.15% in the suburbs. There were 12 fire stations with an accessibility decline ratio over 14% in the city center but only five fire stations with a ratio over 14% in the suburbs. For instance, Hanzhongmen fire station, located in the city center, had the largest accessibility decline ratio during rush hours of all fire stations, largely due to being located in the second most congested Gulou district in terms of the Nanjing travel report in 2019. The report shows that the average travel time in Gulou district in the morning and evening rush hours was 1.97 times of that during non-rush hours. Similarly, Shimenkan fire station is located in the most congested district of Qinhuai district in Nanjing, with an average travel time during rush hour 2.1 times that during non-rush hours. Dongshan and Kaifaqu fire stations had relatively large accessibility decline ratios in the suburbs mainly because the residential buildings around the southeast of the city center have emerged rapidly in recent years and the travel time has significantly increased in the area due to the increase in commuters during rush hours.

## 5. Discussion

**Figure 8:**
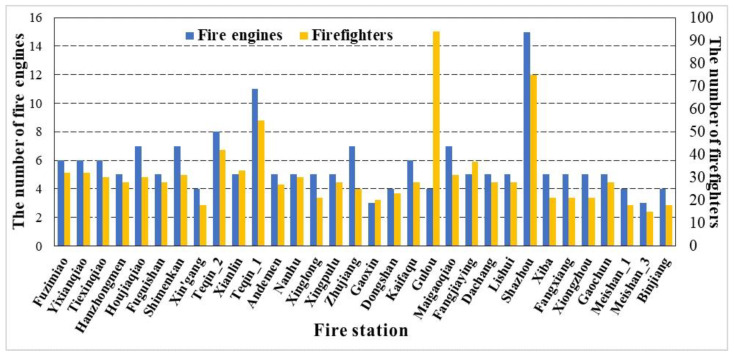
The number of firefighters and fire engines at each fire station in Nanjing, China.

One objective of this study is to measure the spatiotemporal accessibility of fire stations and the potential improvements can be investigated for the fire department. The supply capacity is the key factor for the measurement of accessibility, and the impact of supply capacity on the accessibility of each fire station is therefore discussed. Figure 8 presents the number of firefighters and fire engines at each fire station in Nanjing, China. It shows that the supply capacity of fire stations is related to the accessibility of some fire stations. For instance, Shazhou and Teqin_1 have stronger supply capacity, resulting in higher accessibility. In contrast, Maigaoqiao, Fangjiaying, Shimenkan, Fuzimiao, Hanzhongmen, Xingpulu, Dongshan, Kaifaqu, and Xiongzhou have relatively weaker supply capacity to generate lower accessibility. The improvement in the supply capacity for these fire stations may enhance their accessibility. Hanzhongmen, Fuzimiao, and Shimnekan, located in the city center, are also severely affected by traffic congestion and these fire stations may need to pay more attention to traffic control to enhance fire services. For Dongshan, Kaifaqu, and Xiongzhou, due to their large coverage areas, the improvement in supply capacity will be insufficient, so the adjustment of coverage areas may be required as well.

**Figure 9:**
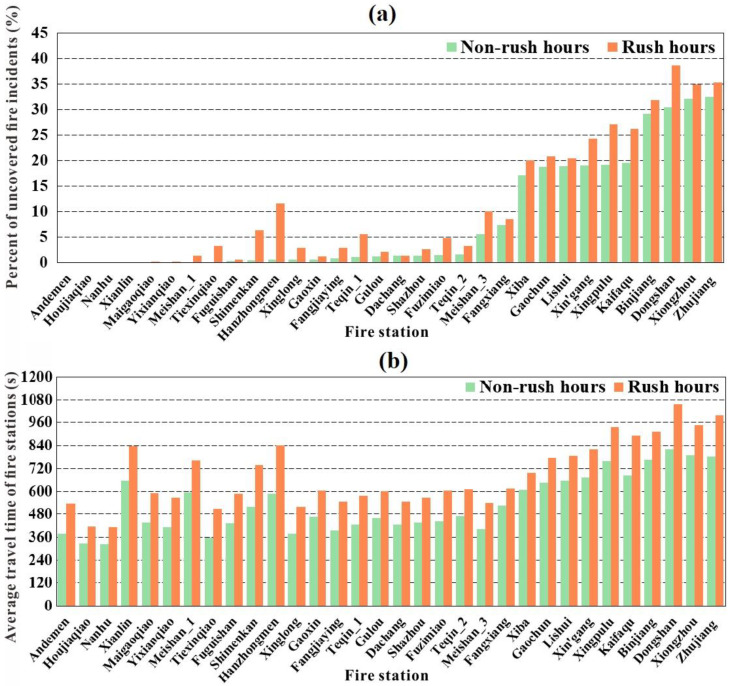
(**a**) Percent of uncovered fire incidents and (**b**) average travel time of fire stations in non-rush and rush hours of 1 day.

Another objective of this study is to explore the spatiotemporal pattern of accessibility and its critical impact factors are discussed. According to the W pattern of accessibility over time, the percent change of uncovered fire incidents and the average travel time change at each fire stations in non-rush and rush hours of 1 day are analyzed. Figure 9a displays the percentage of uncovered fire incidents with travel time over the maximum catchment radius at each fire station in non-rush and rush hours in 1 day. It indicates that most of the fire stations in the suburbs have significantly more uncovered fire incidents than those in the urban center and that the percent of uncovered fire incidents increases considerably in rush hours, especially for the Hanzhongmen and Shimenkan fire stations in the urban center and the Dongshan and Kaifaqu stations in the suburbs. Specifically, all 10 fire stations with more than 20% of fire incidents not being covered were in the suburbs, with the uncovered fire incidents at Hanzhongmen, Shimenkan, Dongshan, and Kaifaqu fire stations increasing by 11.05%, 5.85%, 8.17%, and 6.67% in rush hours, respectively. Hanzhongmen experienced the greatest percent increase in uncovered fire incidents in rush hours and the greatest accessibility decline ratio, as shown in Figure 7. Figure 9b presents the average travel time of fire stations in non-rush and rush hours. It shows that fire stations in the suburbs need more travel time than those in the urban center and that the travel time increase of fire stations in the urban center is relatively higher than in the suburbs during rush hours. Furthermore, Hanzhongmen, Dongshan, Shimenkan, and Kaifaqu are the four fire stations with the greatest travel time increases, 4.3, 4, 3.6, and 3.5 min, respectively, during rush hours, and they are the four with the greatest accessibility decline ratios, as shown in Figure 7.

The results in the case study demonstrated that the developed ST-FC2SFCA can measure the accessibility of fire services in not only space dimension but also time dimension. Compared with existing methods used for only measuring the spatial accessibility of fire services [6,9], the developed ST-FC2SFCA provides an effective solution for examining the temporal variation in the spatial accessibility of fire services. Accordingly, this study can help fire departments to take actions from both spatial and temporal perspectives to improve the real-time accessibility of fire services in terms of the analysis of spatiotemporal accessibility dynamics and the potential impact factors. Several fire stations should improve the supply capacity in terms of the relationship analysis between accessibility and supply capacity. According to the variation of the percent of uncovered fire incidents and travel time in non-rush and rush hours of 1 day, some fire stations should primarily pay attention to the traffic control to reduce the impact of traffic congestion on the accessibility while some fire stations should mainly shrink their coverage areas. This study provides a useful reference for measuring the real-time accessibility of fire services and other public services.

One limitation of this study is that all fire incidents were assumed to distribute uniformly over time in 1 day when measuring the spatiotemporal accessibility due to the information about the time of the fire incidents being unavailable. The spatiotemporal accessibility dynamics of fire services in this study should be considered as an approximation rather than an exact reflection. Therefore, the impact of different fire incident timing on spatiotemporal dynamics of accessibility should be investigated in future works. Further, in addition to the spatiotemporal accessibility dynamics caused by the real-time travel time, there are other possible factors such as the physical environment of fire incidents, which can also affect the accessibility and, therefore, are worthy of future investigation.

## 6. Conclusions

The accessibility measurement of fire services plays an important role in evaluating fire services. We employed the real-time travel time via an online map API to extend an existing spatial accessibility method for measuring the accessibility of fire services in both spatial and temporal dimensions. The extended method was applied in a case study area of Nanjing, China. The main conclusions are as follows:

(1) The overall accessibility of fire incidents and fire stations in Nanjing, China, is uneven, with relatively high accessibility in the southwest and northeast of the city center. Different fire stations require improvement in different resources, such as supply capacity, traffic control, and coverage area adjustment to improve accessibility.
(2) The number of fire incidents with low-level accessibility apparently increases during rush hours in the southeast and north of the city center, and the fire incidents with medium- and high-level accessibility easily change to lower levels due to the influence of traffic congestion. Fire incidents with medium-level accessibility are affected the most. The percent of fire incidents with low-level accessibility during rush hours increases by 10%, whereas fire incidents with medium- and high-level accessibility decrease by about 5%.
(3) The accessibility of fire stations changes over time with an obvious W pattern, where accessibility during rush hours is lower than that at other times, and several fire stations in the city center (e.g., Fuzimiao, Gulou, and Fangjiaying) present an asymmetric W pattern. The accessibilities of fire stations in the whole of Nanjing, city center, and suburbs are 13.41%, 15.97%, and 11.15% lower than those at other times, respectively.
(4) The accessibility decline ratios of fire stations in rush hours are 15.97% and 11.15% in the city center and suburbs, respectively, and there are 12 fire stations in the city center and five fire stations in urban suburbs with an accessibility decline ratio over 14%. The decline ratios are strongly related to the travel time increase and the percent increase in uncovered fire incidents during rush hours, especially for fire stations with a greater decline ratio.

The following suggestions are provided for fire departments to improve fire services in the future: First, several fire stations (e.g., Maigaoqiao, Fangjiaying, Shimenkan, etc.) should improve their relatively low supply capacity to enhance their accessibility. Second, fire stations (e.g., Hanzhongmen, Fuzimiao, Shimenkan, etc.) severely affected by traffic congestion should pay more attention to traffic control, especially for fire incidents with medium-level accessibility. Lastly, fire stations (e.g., Dongshan, Kaifaqu, etc.) with relatively large coverage area need to shrink their coverage areas.

## Data Availability

All data relevant to the study are presented in the article. For further inquiries regarding the reuse of data, please contact the corresponding authors.
